# Anatomical Description of the Main Gyri and Sulci of the Telencephalon of *Alouatta belzebul*

**DOI:** 10.1111/ahe.70104

**Published:** 2026-04-02

**Authors:** Dayane Kelly Sabec Pereira, Fabiano Rodrigues de Melo, Fabiana Cristina Silveira Alves de Melo, Kleber Fernando Pereira, Valcinir Aloisio Scalla Vulcani

**Affiliations:** ^1^ Donaduzzi College Toledo Brazil; ^2^ School of Veterinary Medicine and Animal Science of the Federal University of Goiás Goiânia Brazil; ^3^ Federal University of Viçosa Viçosa Brazil; ^4^ Federal University of Paraná Campus Toledo Toledo Brazil; ^5^ Federal University of Jataí Jataí Brazil

**Keywords:** cortex, howler monkey, primate, telencephalon

## Abstract

The *Alouatta belzebul* is a species of howler monkey, of the Atelidae family and genus *Alouatta*. It is endemic in Brazil and has a separate geographic distribution, with two populations occurring: in the Amazon and in the Atlantic Forest on the coast of Northeast Brazil. The species is classified as ‘vulnerable’, and this is mainly justified by hunting, fragmentation and loss of habitat in tropical forests. Regarding the anatomy of this species, information is scarce and in some aspects such as the brain are non‐existent. The objective of this study is to describe the macroscopy of the main grooves and turns of the *A. belzebul* telencephalon correlating with the degree of encephalisation, the absolute and relative measures of the central groove sinuosity and the straight and sinuous measures of the grooves in different primates. As well as, check the anatomy of the nuclei of the base. Ten *A. belzebul* brains were used, where the gyrations and grooves were dissected. It was found that the brain surface of *A. belzebul* presented with lisencephalic characteristics, similar to the findings with several other species of non‐human primates and differing from species such as *Pan* and *Homo*. In the analysis of the encephalisation index, it was observed that this species is phylogenetically closer to *Sapajus* and *Macaca* and more distant than species such as *Brachyteles* and *Callithrix*, showing expressive cognition and intelligence. Regarding the slope of the central groove, it was observed that in *A. belzebul* the upper end is posterior to the lower end, data that approximate with *Homo*, *Papio* and *Pan* and reveal a large frontal lobe when compared to *Sapajus libidinosus* , showing the maximum brain development in these primates.

## 1 Introduction

The central nervous system (CNS) in *Homo* is anatomically divided into brain and spinal cord, both constitute the neuro‐axis, where the brain is subdivided into brain, cerebellum and brainstem. The brain is further subdivided into telencephalon and diencephalon, embryologically these constitute the forebrain (Machado 1993). The brain is represented by two cerebral hemispheres, a right hemisphere and a left hemisphere that communicate through the corpus callosum located in the medial region of the longitudinal fissure (Meneses 2016).

*Alouatta belzebul* is popularly known as red‐handed howler and, because it is part of the neotropical primate group, it has unique characteristics of the species in relation to motricity, such as having the limbs and trunk adapted for the suspensory behaviour and a truly prehensile long tail (Gregorin 2006). The study of the telencephalon and the protective coatings of the neural system of the *A. belzebul* fits this context and obtaining data becomes important on morphological and, consequently, cortical aspects of this species, as it can provide a basis for neurophysiological interpretations and phylogenetic and evolutionary correlations with other primates. The organisation of sulci and brain gyri related to brain growth determines the degree of cognition, as well as the evolutionary particularities related to the characterisation of the complexity of the species (Testut and Latarjet 1958; Kaas 2006; Isler and Schaik 2009).

In general, each cerebral hemisphere consists of an outer layer of nerve cells where the cerebral grey matter is found, forming the cerebral cortex. In the medial region of the hemispheres is the nuclei or ganglia of the base, organised by the claustrum, putamen, pale medial globe, pale lateral globe and caudate nucleus. The amygdaloid complex and the other juxtacortical limbic nuclei are distributed in the internal structures of the telencephalon, as well as the subcortical white matter composed of the different types of interhemispheric nerve fibres, the association fibres and the projection fibres (Prada 2014).

The evolutionary development of the cerebral hemispheres culminated in the characterisation of Homo's telencephalon, whose more differentiated abilities are due to the development of the neocortex, as well as the size of the central nervous system in relation to the body characterises a degree of encephalisation greatly evolved, especially due to its complex neural network (Jerison 1977; Roth and Dicke 2005).

The macroscopic organisation of homo cerebral hemispheres is composed of frontal lobe, parietal lobe, occipital lobe, temporal lobe and insula lobe. These can be subdivided into poles: frontal, occipital and temporal. Thus, each hemisphere has three surfaces: superolateral, medial, and inferior or basal. The sulci are extensions of the subarachnoid space that are available on the brain surface in order to separate and delimit their gyri. However, it is necessary to consider that the sulci may present different morphologies. They can be continuous or interrupted, long or short, isolated, or connected with other brain sulci. They are classified into four types: limiting sulci, axial sulci, opercular sulci and complete sulci (Meneses 2016; Martin 2013; Machado and Haertel 2014).

Axial sulci develop along the axis of a homogeneous area; the limiting sulci are situated between the cortical areas. The opercular sulci are located between the cortical areas, being differentiated from the limiting sulci that present this separation close to their edges and not related to depth. This difference allows a third functional area to be present. The sulci called complete are those that the depth produces elevations in the walls of the lateral ventricles (Meneses 2016; Martin 2013).

The topographic disposition of brain structures is of prime importance for understanding and describing brain functioning. Sulci, gyri, fissures and ventricular cavities are fundamental for clinical, surgical and imaging contributions. The objective of this work is to describe the macroscopy of the main sulci and gyri of the telencephalon, the degree of inclination of the central sulcus and the straight and sinuous measurements of the encephalic sulci of *A. belzebul* correlating with the degree of encephalisation in different primates.

## 2 Materials and Methods

For this study, ten brains of *A. belzebul* , males and females, adults and lesion‐free, collected during the rescue and rescue period of terrestrial fauna during the implementation activities of the Belo Monte Hydroelectric Power Plant—Brasília‐DF, ruled by IBAMA's process no. 02001.001848/2006‐75 and authorisation no. 473/2014. After the rescue, the animals were frozen and sent to the Laboratory of Human and Comparative Anatomy of the Federal University of Jataí, being kept frozen until the beginning of processing. The experimental procedure was approved by the Ethics Committee on the Use of Animals of the Federal University of Goiás (UFG) (protocol number 083/17).

All animals were weighed and then stored in 10% formaldehyde aqueous solution by intramuscular, subcutaneous and intracavitary injections. The specimens were kept in this solution for at least 72 h. After the fixation period, the brains were carefully taken from the skull, removing the skull cap with the aid of an oscillatory saw (Dremel 3000) in the craniocaudal direction, from the height of the frontal bone to the occipital, in order to maintain brain integrity. The brains were weighed in an Edutec analytical balance model EEQ9003F‐B after the removal and measured with a MTX digital calliper and documented with a digital camera. Macroscopic anatomical descriptions were based on descriptions of human and non‐human primates found in the literature. The brains were measured in the longitudinal and transverse axes.

The sulci of the brains were measured in two ways, first in a straight line and then the sinuosities were considered. The measurements were performed according to the technique described by Pereira‐de‐Paula et al. (2010), in absolute and relative terms (considering the value of the straight measure divided by the curved measure). If the value found for a given sulcus was equal to 1, it means that the sulcus is straight, so it has no curvatures. If the value is close to zero, it indicates greater sinuosity, hence more gyrencephalisation. The straight measurements were obtained by measuring the ends of the sulci directly with the calliper. The sinuous measurements were made with an inextensible line outlining the entire sulcus and then the measurement of the line was obtained with the aid of a calliper.

The encephalisation index was measured by the following ratio: [brain weight/body weight] × 100. Afterwards, statistical analyses of brain measurements were performed, considering mean and standard deviation. For the nomenclature of anatomical structures, veterinary Anatomical Nomina was used.

## 3 Results and Discussion

**FIGURE 1:**
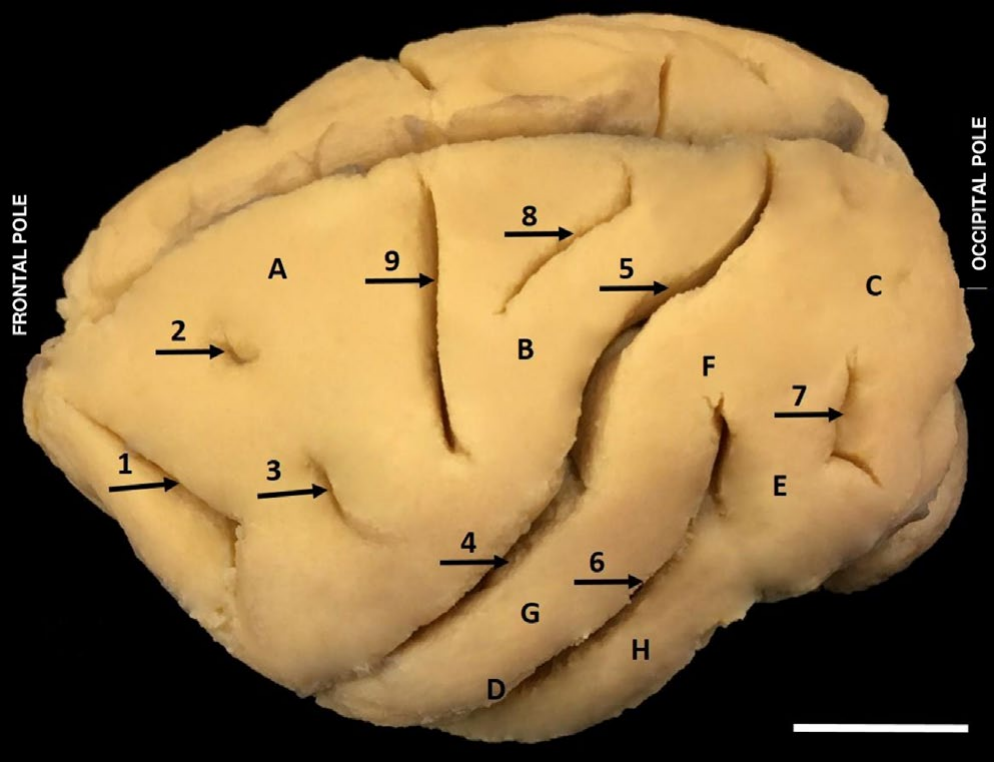
Lateral view of the left hemisphere of *Alouatta belzebul* evidencing the cerebral lobes with their sulci and gyri. (1) Straight sulcus; (2) Superior precentral sulcus; (3) Inferior precentral sulcus; (4) Lateral sulcus; (5) Intraparietal sulcus; (6) Superior temporal sulcus; (7) Lunate sulcus or transverse occipital; (8) Postcentral sulcus; (9) Central sulcus. (A) Frontal lobe; (B) Parietal lobe; (C) Occipital lobe; (D) Temporal lobe; (E) Angular gyrus; (F) Supramarginal gyrus; (G) Superior temporal gyrus; (H) Inferior temporal gyrus (Bar 1 cm).

In the macroscopic anatomical studies of the brain of *A. belzebul* , five cerebral lobes were observed: frontal lobe (A), parietal lobe (B), occipital lobe (C), temporal lobe (D) and insula lobe (E), and most of the brain surface was presented with lissencephalic characteristics (Figure 1).

**FIGURE 2:**
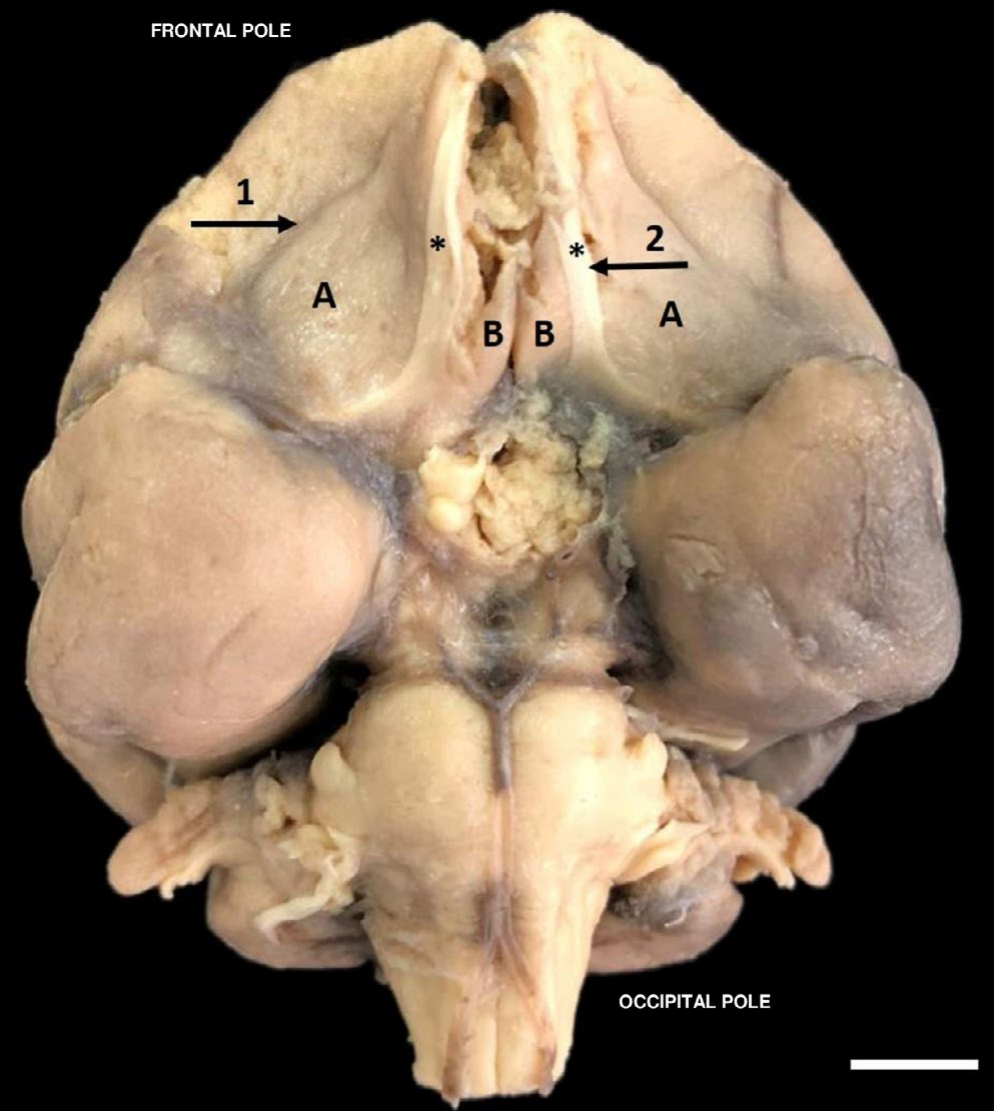
Bottom view of *Alouatta belzebul* brain showing cerebral sulci. (1) Orbital sulcus; (2) Olfactory sulcus; (A) Orbital gyrus; (B) Straight gyrus; (*) Olfactory nerve. (Bar 1 cm).

The frontal lobe of *A. belzebul* has as its anterior limit the frontal pole and as its posterior limit the central sulcus (9), as its superior limit the frontal lobe has the longitudinal fissure of the telencephalons and as its inferior limit the lateral sulcus (4) (Figure 1). In this lobe, the presence of three cerebral sulci was observed in the cranial lateral face of the frontal lobe: straight sulcus (1), superior precentral sulcus (2) and inferior precentral sulcus (3). The straight sulcus in *A. belzebul* presented a long and deep path. The superior precentral sulcus presented a short path when compared with the inferior precentral sulcus, which has its path a little longer and with a sharper curvature. On the inferior face of the frontal lobe, the presence of the olfactory sulcus (1) was observed, which medially delimits the straight gyrus and, laterally to this sulcus, the orbital sulci that delimit the orbital gyrus and form the entire frontobasal surface were observed (Figure 2).

The presence of five cerebral lobes and several sulci distributed throughout the regions of the telencephalon was observed in *A. belzebul* , as well as the presence of the longitudinal fissure of the telencephalon which divides the telencephalon into the right and left hemispheres and are internally connected by the corpus callosum. The brain surface showed mostly lissencephalic characteristics, however, in some areas such as the parietal and temporal lobes, sharper curvatures were observed. These data corroborate the findings in brains of *Ateles geoffrovi* (ornate spider monkey) (Conolly 1936; León et al. 2009), *Alouatta seniculus* (jurua red howler) (Conolly 1936), *Macaca fascicularis* (Connolly 1950; Kashima et al. 2008), *Macaca mulatta* (rhesus monkey) (Conolly 1936; Geist 1930), *Papio cynocephalus* (baboons) (Conolly 1936; Turner 1890), *Callith penrixicillata* (Conolly 1936; Rylands and Mendes 2008), *Saimiri ustus* (Connolly 1950; Goldschmidt et al. 2009) and *Brachyteles arachnoides* (Conolly 1936; Milton 1984; Mendes et al. 2008). *Sapajus libidinosus* showed gyrencephalic characteristics, phylogenetically approaching to *Pan* and *Homo* (Pereira‐de‐Paula et al. 2010; Fragaszy et al. 2004, 2013). From the phylogenetic point of view, the hippocampus sulcus was the first one to appear, limiting the archcortex, while the second was the rhinal sulcus, separading the archcortex from the neocortex (Abreu et al. 2021). Both sulci were observed in *A. belzebul* and are present in non‐human.

**FIGURE 3:**
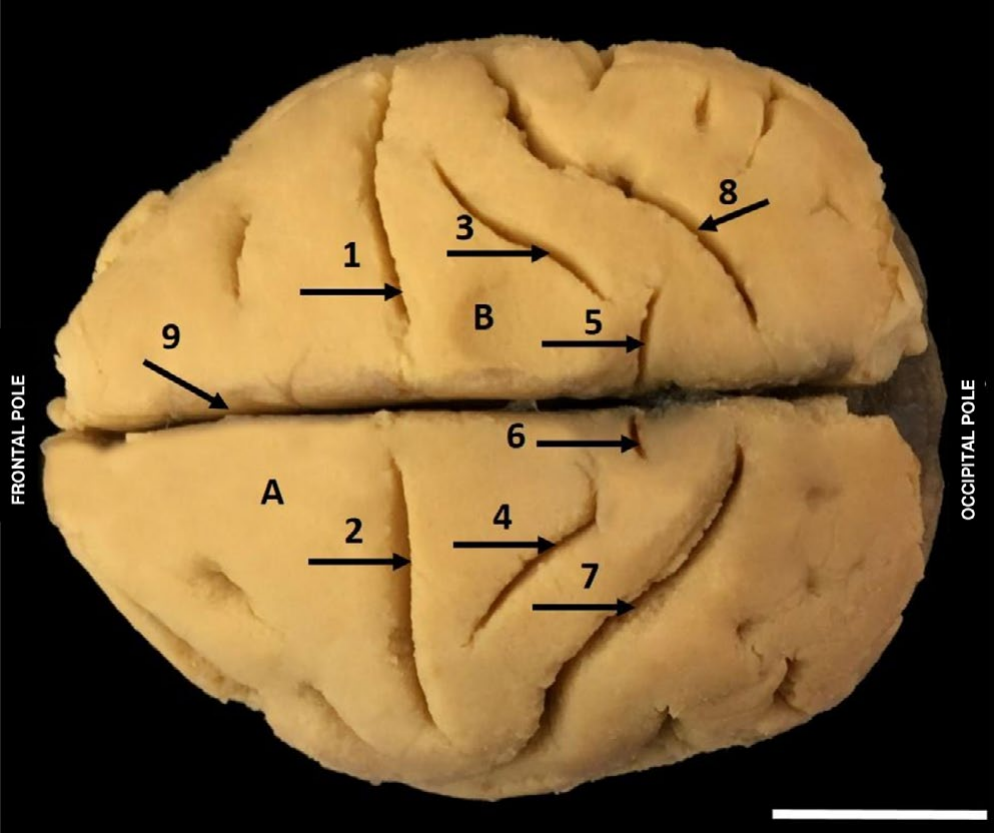
Superior view of the left and right hemispheres of *Alouatta belzebul* showing cerebral sulci. (1) Central sulcus right hemisphere; (2) Central sulcus left hemisphere; (3) Postcentral sulcus right hemisphere; (4) Postcentral sulcus left hemisphere; (5) Parieto‐occipital sulcus right hemisphere; (6) Parieto‐occipital sulcus left hemisphere; (7) Intraparietal sulcus left hemisphere; (8) Intraparietal sulcus right hemisphere; (9) Longitudinal fissure of the brain (A) Precentral gyrus; (B) Postcentral gyrus. (Bar 1 cm).

The central sulcus in *A. belzebul* separates the superior and inferior precentral gyrus from the postcentral sulcus. In the superior pole, the central sulcus penetrates the medial face of the hemisphere in the paracentral gyrus region, and inferiorly, does not reach the lateral sulcus, presenting a straight downward path, in such a way as to characterise the superior and inferior connection between the two gyri (Figure 3).

The superior precentral sulcus in *A. belzebul* was identified as short and shallow, data that corroborate the findings in *B. arachnoides* and *A. seniculus* and in *Ateles geoffroyi* , this sulcus presents a long and arched path forming a horizontal branch until near the central sulcus (Conolly 1936; León et al. 2009; Milton 1984). In *S. libidinosus* , this sulcus was identified as a superior longitudinal sulcus with rectilinear and deep morphology with discrete curvature (Pereira‐de‐Paula et al. 2010). In *Callithrix penicillata* and *Galago senegalensis senegalensis* , the frontal lobe is devoid of sulci, which hinders the delimitation of the lobes and in *S. ustus* the central sulcus is poorly developed (Abreu et al. 2021; Markowitsch et al. 1890). In *M. mulatta* , *P. cynocephalus* and *Pan troglodytes* , the superior precentral sulcus is evidenced by rather irregular morphology, usually in an oblique position (Connolly 1950). In the *M. fascicularis* species, the precentral sulcus is present; however, with a different nomenclature, called the superior arched sulcus (Kashima et al. 2008; Fukunishi et al. 2006). In *Homo*, the precentral sulcus is formed by the superior and inferior frontal gyri, which are longitudinally arranged and separated by the superior and inferior frontal sulci, also horizontally arranged; the precentral gyrus is responsible for the primary motor activity in this species (Meneses 2016; Van De Graaff 2003).

The inferior precentral sulcus was identified in *A. belzebul* with a slightly longer path when compared to the superior precentral sulcus and with a sharper curvature. In *S. libidinosus* species, this sulcus was called the inferior longitudinal sulcus with rectilinear and deep morphology and with discrete curvature (Pereira‐de‐Paula et al. 2010). In *C. penicillata* , *G. senegalensis senegalensis* and *S. ustus* , the inferior precentral sulcus is absent (Rylands and Mendes 2008; Goldschmidt et al. 2009; Abreu et al. 2021; Markowitsch et al. 1890). In *M. mulatta* , *P. cynocephalus* , *P. troglodytes* , *B. arachnoides* , *A. seniculus* and *A. geoffroyi* (Conolly 1936; Geist 1930; Preuss and Goldman‐Raki 1991), this sulcus is present, and it can also be called the inferior arched, presenting concave morphology in the cranial direction with horizontal branching. In *Homo*, this sulcus is called the inferior frontal sulcus, delimiting the inferior frontal gyrus that is divided by branches of the sylvian fissure into three parts: orbital part, triangular part and opercular part. These last two parts are responsible for the motor area of language (Broca's area) in the dominant hemispheres (Meneses 2016; Machado and Haertel 2014).

In the frontal pole of the telencephalon of the *A. belzebul* , a straight sulcus was observed, with a long and deep downward path, its origin is close to the longitudinal fissure of the telencephalon and ends near the lateral sulcus. These data are in accordance with the results for *A. geoffroyi* , *A. seniculus* , *P. cynocephalus* , *P. troglodytes* (León et al. 2009; Connolly 1950; Platas‐Neri et al. 2019) and differ from those found for *S. libidinosus* , because this sulcus does not delimit the frontal lobe gyri and it receives the inferior longitudinal sulcus nomenclature (Pereira‐de‐Paula et al. 2010) in this species. In the *M. mulatta* species, this sulcus was called frontal sulcus (Geist 1930) and in *M. fascicularis* , it was called the main sulcus (Kashima et al. 2008; Fukunishi et al. 2006). In *C. penicillata* , *G. senegalensis senegalensis* and *S. ustus* this sulcus is absent (Abreu et al. 2021; Markowitsch et al. 1890). In *Brachyteles arachnoids* (Conolly 1936; Mendes et al. 2008) and in *Homo* (Meneses 2016; Martin 2013), this sulcus is described as the inferior frontal sulcus, delimiting a larger part of the inferior frontal gyrus area.

In the frontobasal (Figure 2) or orbital portion of the frontal lobe of *A. belzebul* , it is observed the olfactory sulcus where the olfactory bulb and the olfactory tract are lodged. Medially to this sulcus, the straight gyrus is located and, laterally, the orbital gyri that form most of the frontobasal surface. These data have not been reported in the literature in other non‐human primates. In *Homo*, corroborate the findings in this species, and it has as function the initiation of voluntary motor impulses for skeletal muscle movements, sensory experiences analysis and responses related to personality, as well as responses related to memory, emotions, reasoning, judgement, planning and verbal communication (Van De Graaff 2003; Noureldine 2019). This fact may justify the great mobility that the studied species presents by having a prehensile tail and fast moves with the limbs to move around in nature.

In the parietal lobe of the *A. belzebul* , the central sulcus, superior limit to longitudinal fissure of the telencephalon and posterior limit of the parieto‐occipital, was observed as the anterior limit. In this lobe, the central and postcentral sulcus delimit the postcentral gyrus, as well as the lateral sulcus separates the parietal lobe from the temporal lobe, delimiting the supramarginal and the angular gyrus (Figure 1). The lateral sulcus in this species is continuous with the intraparietal sulcus.

In the parietal lobe of *A. belzebul* , it was verified the presence of the non‐segmented postcentral sulcus with moderate depth and with a slight concave curvature turned to the longitudinal fissure of the telencephalon with rectilinear downward path until near the central sulcus, delimiting the postcentral gyrus. In *S. libidinosus* , this sulcus is relatively short and attaches caudally to the lunate sulcus (Pereira‐de‐Paula et al. 2010). In *A. geoffroyi* , *A. seniculus* , *P. cynocephalus* and *P. troglodytes* , the postcentral sulcus is segmented into superior and inferior postcentral sulcus. In these last two species mentioned, the union of this sulcus to the intraparietal sulcus can often occur (Connolly 1950). In the species *C. penicillata* , *G. senegalensis senegalensis* and *S. ustus* , this sulcus is absent (Abreu et al. 2021). In *B. arachnoides* (Milton 1984; Mendes et al. 2008), *M. mulatta* (Geist 1930; Preuss and Goldman‐Raki 1991) and *M. fascicularis* (Kashima et al. 2008; Fukunishi et al. 2006), the postcentral sulcus is unique with a short path. In *Homo*, this sulcus is located on the superolateral surface, delimiting the postcentral gyrus, extending in parallel to the central sulcus (Meneses 2016; Machado and Haertel 2014). The parietal lobe in *Homo* responds to somesthetic stimuli, besides performing the function in the understanding of speech and in the articulation of thoughts and emotions, and also in the interpretation of textures and shapes of objects when manipulated (Van De Graaff 2003; Noureldine 2019).

In *A. belzebul* , it was observed that the parieto‐occipital sulcus separates the parietal lobe from the occipital lobe, both at the superomedial edge and on the superolateral surface. This sulcus is located posterior to the postcentral sulcus and prior to the intraparietal sulcus, with a rectilinear and deep path in the superolateral part of the surface and a shallow path in the superomedial part. These data were also found in *B. arachnoides* , *A. geoffroyi* and *A. seniculus* (Connolly 1950; Milton 1984; Mendes et al. 2008; Platas‐Neri et al. 2019); however, in *Ateles* and *Brachyteles*, this sulcus follows its caudal path and presents an accessory parieto‐occipital sulcus. In *S. libidinosus* (Pereira‐de‐Paula et al. 2010), the parieto‐occipital sulcus joins the lunate sulcus, forming a continuous path on the superolateral surface; in *S. ustus* (Goldschmidt et al. 2009; Abreu et al. 2021), this sulcus joins in the medial part with the lateral sulcus in a continuous path. In *C. penicillata* and *G. senegalensis senegalensis* , this sulcus is absent (Turner 1890; Kanagasutheram and Mahran 1960). In *P. cynocephalus* , *P. troglodytes* (Conolly 1936; Swindler and Wood 1973), *M. mulatta* (Geist 1930) and *M. fascicularis* (Kashima et al. 2008; Fukunishi et al. 2006), this sulcus is present not joining with the calcarine sulcus. In *Homo*, this sulcus is deep, and it is in the medial part forming a right angle with the calcarine sulcus (Machado and Haertel 2014), however, they are not united, being separated by the calcarine sulcus gyri (Ribas 2010).

**FIGURE 4:**
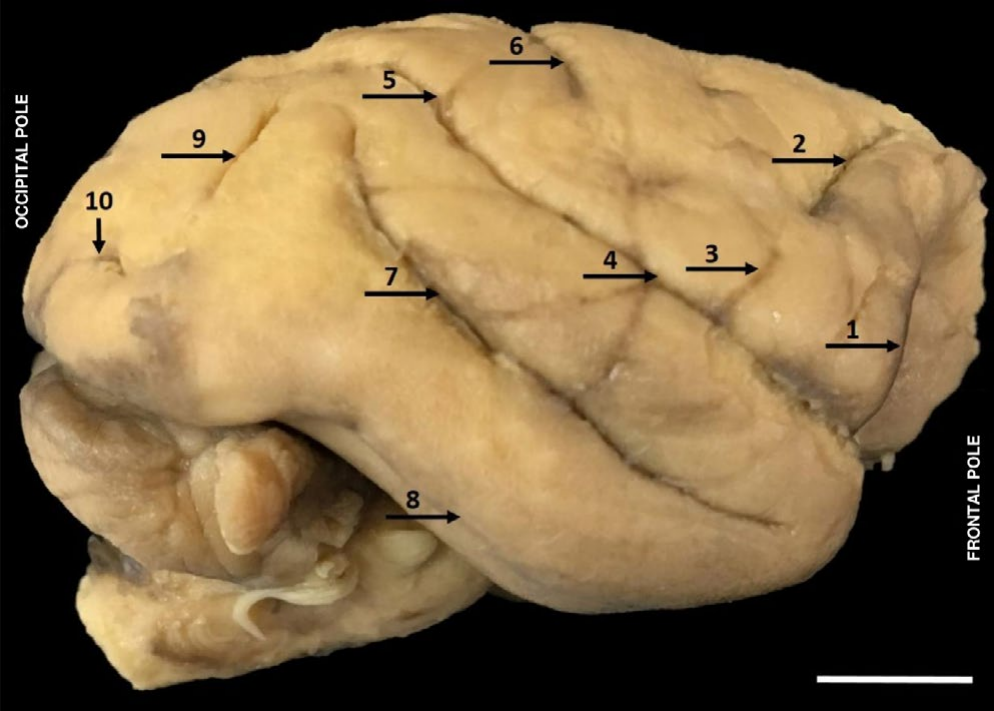
—Lateral view of the right hemisphere of *Alouatta belzebul* showing cerebral sulci. (1) Orbital sulcus; (2) Superior precentral sulcus; (3) Inferior precentral sulcus; (4) Lateral sulcus; (5) Intraparietal sulcus; (6) Central sulcus; (7) Superior temporal sulcus; (8) Inferior temporal sulcus; (9) Lunate sulcus; (10) Retro‐calcarine sulcus. (Bar 1 cm).

In the temporal lobe of the *A. belzebul* , the superior and inferior temporal sulci were observed, delimiting the superior and the inferior temporal gyri. The superior temporal gyrus is delimited by the lateral sulcus and the superior temporal sulcus beginning a few millimetres from the temporal pole and following a continuous upward oblique path in the parietal lobe, where it goes around resulting in the supramarginal gyrus and the angular gyrus. The inferior temporal gyrus is delimited by the superior temporal sulcus and inferior temporal sulcus, which presented a short path delimiting the inferior temporal gyrus with the inferior part of the brain (Figure 4).

In *A. belzebul* , the presence of the lateral sulcus (4) (Figure 4) was observed, separating the frontal and parietal lobe from the temporal lobe, where in this species, the lateral sulcus is deep and sinuous, joining the intraparietal sulcus (5) (Figure 4), forming a continuous path. The intraparietal sulcus divides the parietal superolateral surface into the inferior and superior parietal lobes; in the inferior lobe, the supramarginal gyrus is observed, which is curved close to the lateral sulcus at the distal end; in the central part the angular gyrus is observed, which is related to the distal end of the superior temporal sulcus. These data are similar to the ones found in *A. geoffroyi* ; the difference is in the path of this sulcus which in this species is close to the lunate sulci, and in *A. seniculus* , the path of the lateral sulcus ends in a bifurcation (Conolly 1936). In *S. libidinosus* , this sulcus presents itself with a posterior inclination and does not join the central sulcus, presenting a deviation and ends its path in the parietal lobe (Pereira‐de‐Paula et al. 2010). In *B. arachnoides* (Connolly 1950; Milton 1984; Mendes et al. 2008), the description is similar to *S. libidinosus* , except for presenting two deviations before finishing its path in the parietal lobe. In the *C. penicillata* (Rylands and Mendes 2008) and *G. senegalensis* (Markowitsch et al. 1890) species, the lateral sulcus begins in the olfactory stria, ascends in the posterior direction and ends on the dorsolateral surface of the brain. In *S. ustus* , the lateral sulcus has an ascending path through the dorsolateral surface with a deviation and ends close to the longitudinal fissure of the telecephalon (Goldschmidt et al. 2009; Abreu et al. 2021). In *P. cynocephalus* , *P. troglodytes* (Conolly 1936), *M. mulatta* (Geist 1930) and *M. fascicularis* (Kashima et al. 2008; Fukunishi et al. 2006), the lateral sulcus has an ascending path to the parietal lobe; what differs these species is the variation in the curvature degree that is anatomically characterised gyrencephalic.

The superior temporal sulcus was observed in *A. belzebul* inferior to the lateral sulcus, near the temporal pole and follows an ascending oblique path to the parietal lobe, contouring the supramarginal and angular gyri. These data corroborate the findings in *M. mulatta* (Geist 1930), *P. cynocephalus* (Conolly 1936), *M. fascicularis* (Kashima et al. 2008; Fukunishi et al. 2006) and *Homo* (Meneses 2016) which present this sulcus well‐developed. In *G. senegalensis senegalensis* (Kanagasutheram and Mahran 1960), this sulcus is absent. In *C. penicillata* and *S. ustus* , the superior temporal sulcus is not located posterior to the lateral sulcus; in *Callithrix*, this sulcus is slightly more sinuous and shorter when compared *to Saimiri* (Abreu et al. 2021). In *S. libidinosus* , this sulcus meets with the lateral sulcus in its caudal part, as well as in *B. arachnoides* and *A. geoffroyi* (León et al. 2009; Milton 1984). In *P. troglodytes* (Connolly 1950; Swindler and Wood 1973), the posterior part of the superior temporal sulcus joins the lunate sulcus.

The inferior temporal sulcus in *A. belzebul* presents a short, rectilinear, and shallow path, delimiting the inferior temporal gyrus with the inferior portion of the brain. These data are similar to those found in *S. libidinosus* (Pereira‐de‐Paula et al. 2010), *B. arachnoides* and *A. geoffroyi* (Conolly 1936; León et al. 2009; Milton 1984). In *P. troglodytes* , this sulcus is developed; however, it presents segmentations in its path (Swindler and Wood 1973), data that approaches Homo (Meneses 2016; Kiernan 2003). In *M. mulatta* (Geist 1930), *P. cynocephalus* (Conolly 1936) and *M. fascicularis* (Kashima et al. 2008; Fukunishi et al. 2006) species, this sulcus is present delimiting the inferior temporal gyrus and occipitotemporal gyrus. In the species *C. penicillata* , *G. senegalensis* and *S. ustus* , this sulcus is absent (Abreu et al. 2021). In *Homo*, the temporal lobe performs the function of interpreting auditory sensations and storing auditory and visual memory (Martin 2013; Machado and Haertel 2014).

**FIGURE 5:**
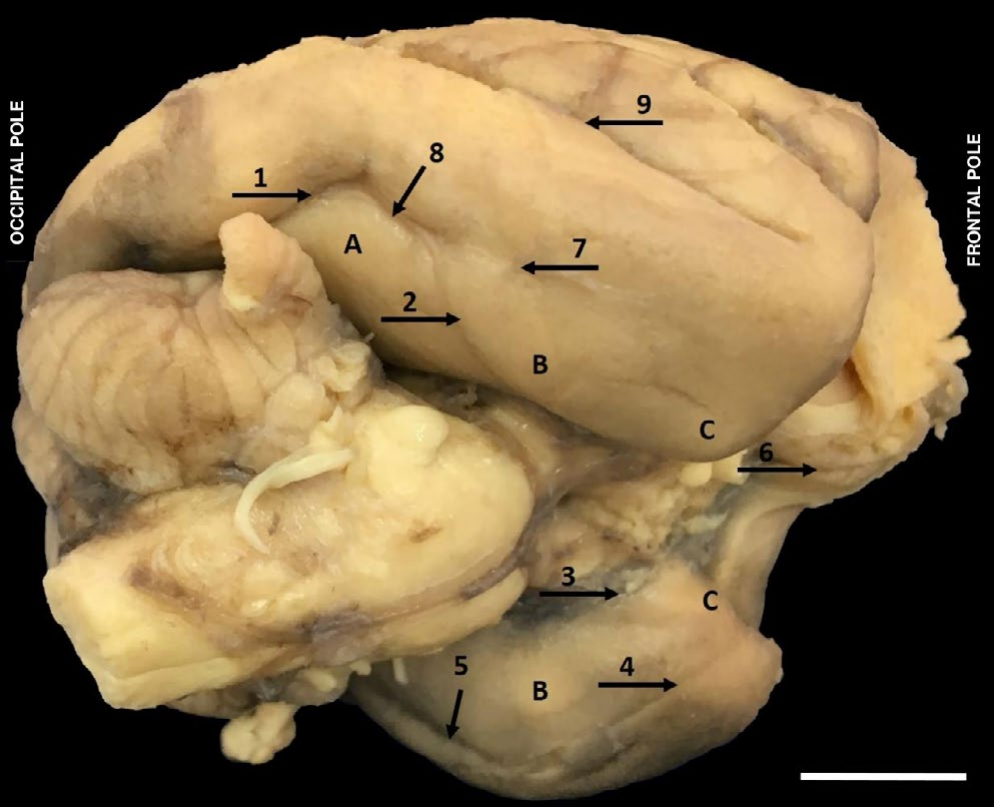
Inferior view of *Alouatta belzebul* brain to show cerebral sulci. (1) Collateral sulcus; (2) Occipitotemporal sulcus; (3) Hippocampal sulcus; (4) Rhinal sulcus; (5) Retro‐calcarine sulcus; (6) Orbital sulci; (7) Inferior temporal sulcus; (8) Inferior occipital sulcus; (9) Superior temporal sulcus; (A) Occipitotemporal or lingual gyrus; (B) Parahippocampal gyrus; (C) Uncus. (Bar 1 cm).

In *A. belzebul* , the rhinal sulcus is located in the inferior portion of the temporal lobe, with a parallel path to the hippocampus sulcus and a continuous path with the retro‐calcarine sulcus, delimiting the anterior part of the parahippocampal gyrus (Figure 5). These data are similar to those described for *S. libidinosus* (Pereira‐de‐Paula et al. 2010), *A. seniculus* (Connolly 1950), *M. mulatta* (Geist 1930), *M. fascicularis* (Kashima et al. 2008; Fukunishi et al. 2006), *P. cynocephalus* and *P. troglodytes* (Connolly 1950; Swindler and Wood 1973), *A. geoffroyi* , *B. arachnoides* (Connolly 1950) and *Homo* (Meneses 2016; Machado and Haertel 2014). In *G. senegalensis senegalensis* (Preuss and Goldman‐Raki 1991; Kanagasutheram and Mahran 1960), *S. ustus* and *C. penicillata* (Rylands and Mendes 2008; Abreu et al. 2021), the rhinal sulcus has a shallow characteristic separating the piriform lobe from the temporal lobe.

The occipital lobe of the *A. belzebul* is delimited in its anterior portion by the parieto‐occipital sulcus at the superomedial edge of the hemispheres and ends in the occipital pole, presenting lissencephalic characteristics (Figure 4). On the medial surface of the occipital lobe, the gyri and sulci are highly defined; its main sulcus is the calcarine, which, in this species, is subdivided into paracalcarine and retro‐calcarine (Figure 5).

The lingual gyrus is superiorly delimited by the collateral sulcus and inferiorly by the hippocampal sulcus, bordered by the inferior occipital sulcus and the occipitotemporal sulcus (Figure 5). In the inferior part of the brain of *A. belzebul* , the rhinal sulcus was identified, which presents itself continuous to the retro‐calcarine sulcus and delimits the parahippocampal gyrus. Also in the findings, the short path inferior occipital sulcus joining continuously with the collateral sulcus and the occipitotemporal sulcus was identified (Figure 5). The uncus is an anatomical structure with a function related to the emotional system. In *A. belzebul* , it was identified between the hippocampal sulcus and the rhinal sulcus, important for correlations with the limbic system.

This occipital lobe is the one with the highest morphological variations discussed within the non‐human primates found in the literature. In *A. belzebul* , the lunate sulcus was observed between the intraparietal sulcus and the calcarine sulcus; it is short, rectilinear and does not separate the lobes, having no relation to any other sulcus. These findings were also observed in *A. seniculus* (Conolly 1936). In *C. penicillata* , *G. senegalensis* this sulcus is absent. In *S. ustus* , it is underdeveloped (Abreu et al. 2021). In *S. libidinosus* (Pereira‐de‐Paula et al. 2010), the lunate sulcus has a continuous path with the postcentral sulcus. In *B. arachnoides* and *A. geoffroyi* (Milton 1984; Platas‐Neri et al. 2019), this sulcus presents variations in its path; in *Brachyteles* it can join with the lateral sulcus, while in *Ateles* it can join with the intraparietal sulcus, data similar to those found for *P. cynocephalus* (Conolly 1936) and *P. troglodytes* (Swindler and Wood 1973). In *Homo*, the lunate sulcus is present and is located on the superomedial edge, with the preoccipital incisure located on the inferolateral edge, near the occipital pole (Meneses 2016; Armstrong et al. 1991; Tamraz and Comair 2000).

The occipital lobe of the *A. belzebul* is limited to the temporal lobe through the collateral sulcus in a continuous anterior path with the inferior occipital and occipitotemporal sulcus. These delimit the occipitotemporal or lingual gyrus, which in the anterior path continues with the parahippocampal gyrus that configures the basal temporal‐occipital surface portion (Figure 5). In the posterior part, it joins the calcarine sulcus. These data are similar to those found for *S. ustus* (Abreu et al. 2021), *S. libidinosus* (Pereira‐de‐Paula et al. 2010), *A. seniculus* (Conolly 1936), *M. mulatta* (Geist 1930), *M. fascicularis* (Kashima et al. 2008; Fukunishi et al. 2006), *P. cynocephalus* and *P. troglodytes* (Conolly 1936; Swindler and Wood 1973), *A. geoffroyi* and *Brachyteles arachnoids* (Connolly 1950), except *S. ustus* (Abreu et al. 2021) because in this last one, the collateral sulcus does not join to the calcarine sulcus. In *Homo*, the collateral sulcus is well developed with origin in the occipital pole and anterior path, followed by the calcarine sulcus, hippocampus sulcus and medial occipitotemporal gyrus (lingual gyrus) and parahippocampal gyrus (Meneses 2016; Machado and Haertel 2014; Ribas 2010). The occipitotemporal sulcus is absent in *B. arachnoides* (Conolly 1936), *G. senegalensis* (Preuss and Goldman‐Raki 1991; Kanagasutheram and Mahran 1960), *S. ustus* and *C. penicillata* (Abreu et al. 2021).

In the occipital lobe of *A. belzebul* , it was observed the inferior, short, rectilinear, deep and continuous occipital sulcus with the collateral sulcus and the occipitotemporal sulcus, delimiting the occipitotemporal gyrus. These findings have also been described for *A. geoffroyi* (León et al. 2009) and *A. seniculus* (Conolly 1936), which differentiates them is the sulcus size that is larger for *Ateles*, as well as for *P. cynocephalus* , *B. arachnoides* (Connolly 1950; Milton 1984; Mendes et al. 2008) and *M. fascicularis* (Kashima et al. 2008; Fukunishi et al. 2006). In *P. troglodytes* (Connolly 1950; Swindler and Wood 1973), the inferior occipital sulcus for having a variable path, joining to the middle temporal sulcus or to the occipitotemporal sulcus or still not joining any other sulcus. In *C. penicillata* , *G. senegalensis* and *S. ustus* , this sulcus is absent (Abreu et al. 2021). In *Homo*, the occipital sulci delimit the superior, middle and inferior occipital gyri (Meneses 2016; Machado and Haertel 2014; Ribas 2010).

In *A. belzebul* , the calcarine sulcus has its origin on the surface of the occipital pole, inferior to the lunate sulcus and follows a descending path to the posteromedial part of the occipital lobe; it is presented as long, shallow and with a slight sinuosity, presenting two smaller sulci, the retro‐calcarine, in the posterior portion and the paracalcarino anterior to the parieto‐occipital sulcus. These findings are similar to those described for *A. geoffroyi* and *A. seniculus* (Conolly 1936). In *G. senegalensis* (Preuss and Goldman‐Raki 1991; Kanagasutheram and Mahran 1960) in addition to these two sulci mentioned above, the pre‐calcarine sulcus with origin near the hippocampus sulcus was described. In *Callithrix jacchus* (Reis and Erhart 1979; Sawada et al. 2014), *C. penicillata* , *S. ustus* (Rylands and Mendes 2008; Goldschmidt et al. 2009; Abreu et al. 2021), *S. libidinosus* (Pereira‐de‐Paula et al. 2010), *M. mulatta* (Geist 1930; Preuss and Goldman‐Raki 1991), *M. fascicularis* (Kashima et al. 2008; Fukunishi et al. 2006), *B. arachnoides* , *P. cynocephalus* , *P. troglodytes* (Conolly 1936; Swindler and Wood 1973), this sulcus has a path in the occipital pole and is divided into two branches: one superior and one inferior. In *Homo*, the calcarine sulcus presents an arched path to the occipital pole, with origin inferior to the splenium of the corpus callosum (Machado and Haertel 2014; Noureldine 2019; Kiernan 2003).

In *A. belzebul* , the subparietal sulcus is absent, data corroborating the findings for *A. geoffroyi* , *B. arachnoides* , *A. seniculus* (Conolly 1936), *C. penicillata* , *G. senegalensis senegalensis* (Turner 1890; Kanagasutheram and Mahran 1960), other than *S. libidinosus* (Pereira‐de‐Paula et al. 2010), *S. ustus* (Abreu et al. 2021); *M. mulatta* (Geist 1930), *M. fascicularis* (Kashima et al. 2008; Fukunishi et al. 2006), *P. cynocephalus* , *P. troglodytes* (Conolly 1936; Swindler and Wood 1973) and *Homo* (Meneses 2016), in which this sulcus is present.

The parahippocampal gyrus is medially delimited by the hippocampal sulcus located at the inferior portion of the diencephalic region; in its posterior part, it is also constituted as a continuation anterior to the lingual gyrus, which is located under the calcarine sulcus. In the incisural space of the parahippocampal gyrus laterally to the mesencephalic pedicle, the uncus is formed (Figure 5).

In *A. belzebul* , the hippocampus sulcus was identified near the splenium of the corpus callosum, where it is joined with the corpus callosum sulcus and with the calcarine sulcus, following a path towards the temporal lobe, delimiting the parahippocampal gyrus and the uncus. These data are also found in *S. libidinosus* (Pereira‐de‐Paula et al. 2010), *A. geoffroyi* , *B. arachnoides* , *A. seniculus* (Conolly 1936; León et al. 2009; Milton 1984; Mendes et al. 2008; Platas‐Neri et al. 2019), *M. mulatta* (Geist 1930), *M. fascicularis* (Kashima et al. 2008; Fukunishi et al. 2006), *P. cynocephalus* , *P. troglodytes* (Conolly 1936; Swindler and Wood 1973), *S. ustus* , *C. penicillata* (Rylands and Mendes 2008; Goldschmidt et al. 2009; Abreu et al. 2021). In *G. senegalensis* (Preuss and Goldman‐Raki 1991; Kanagasutheram and Mahran 1960), this sulcus has a path from the posterior region to the anterior region reaching the tubercle of the hippocampus, a structure similar to the uncus in humans. In *Homo*, this sulcus differs from non‐human primates by being continuous only with the corpus callosum sulcus (Meneses 2016; Martin 2013; Noureldine 2019). The occipital lobe in *Homo* has as function the integration of the eye focusing movements, the correlation of visual images with previous visual experiences and other sensory incentives and conscious perception of vision (Van De Graaff 2003).

**FIGURE 6:**
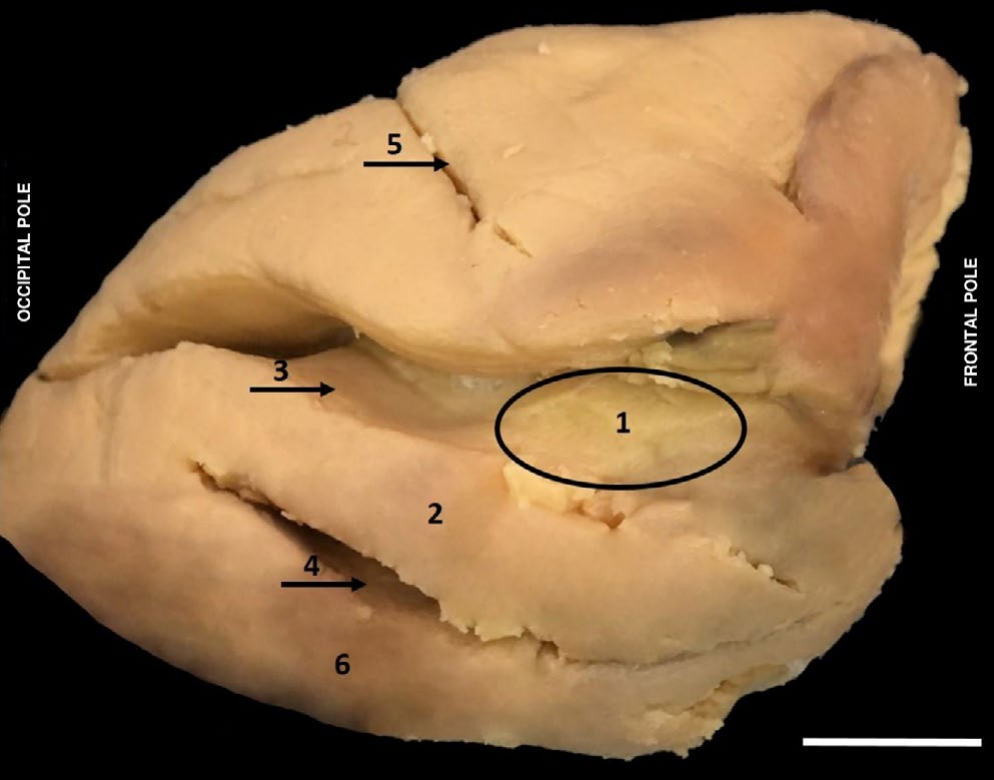
Lateral view of the right hemisphere of *Alouatta belzebul* with partial seizure of the temporal lobe showing cerebral sulci and gyri. (1) Insula wolf (circle); (2) Superior temporal gyrus; (3) Lateral sulcus; (4) Superior temporal sulcus; (5) Central sulcus; (6) Inferior temporal gyrus (Bar 1 cm).

The insula lobe of the *A. belzebul* consists of an invaginated cortical surface of lissencephalic anatomical characteristic, where a circular sulcus was observed skirting a single longitudinal gyrus (Figure 6). In mammals, this lobe is part of the limbic system and coordinates emotions. Structures that constitute this system, such as cingulate gyrus, parahippocampal gyrus, hippocampus, thalamus, hypothalamus and claustrum (Prada 2014), were observed in *A. belzebul* . It is important to highlight that the scientific literature describes that the function of the claustrum is not clear, both in humans and non‐human primates. However, in a study conducted by Redouté et al. (2005) in *Homo*, they verified which region of the telencephalon would be directly linked to the visual sexual stimulus, believing that the blood flow of the visual cortex would be increased and revealed that claustrum activity is related to the intensity of sexual desire and to the arousal.

In research conducted with stimulation of the limbic system structures in non‐human primates, MacLean (1990) unfolded the limbic ring in two strands, where the inferior part would be linked to self‐preservation mechanisms (mainly involving the hypothalamus and the amygdala), and the superior part would correspond to the perpetuation mechanisms of the species (involving the hippocampus, the cingulate gyrus and the septal area). As a result, he inferred that the instinctive motivation of self‐preservation is more primitive and somewhat more intense than the perpetuation of the species.

One of the first studies on the limbic system in Rhesus monkey was conducted with Klüver and Bucy (1937) and consisted of the bilateral removal of the anterior part of the temporal lobes with consequent injury of the hippocampus, parahippocampal gyrus and amygdaloid body, resulting in the pathological process known as Klüver and Bucy syndrome, in which the species began to have domestication, visual agnosia (inability to recognise danger in a situation that previously caused escape reactions, such as the presence of snakes and scorpions), oral tendency (they take everything to the mouth, including scorpions) and hypersexual tendency (continuously try the sexual act and masturbate).

In *Homo*, the insula lobe is gyrencephalic, with the presence of long and short gyrus delimited by a central sulcus and a circular sulcus. It exercises memory function, integration with other brain activities such as the limbic system related to the expression of behaviour accompanied by emotional manifestations in this species (Brandão 2004).

**FIGURE 7:**
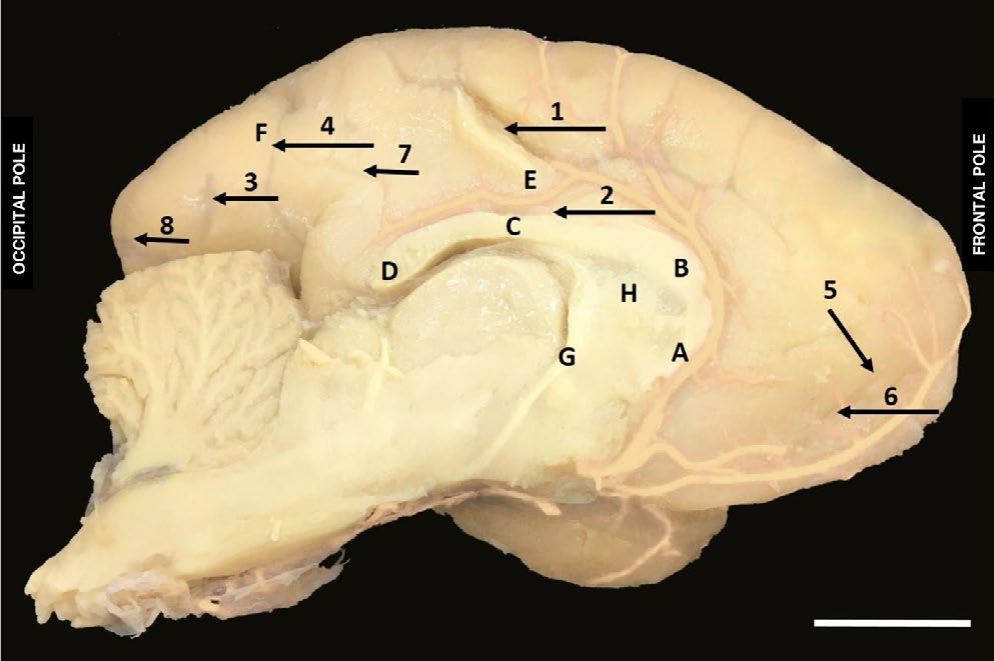
Medial view in sagittal section of the brain of *Alouatta belzebul* . (1) Cingugulus sulcus; (2) Corpus callosum sulcus; (3) Calcarine sulcus; (4) Parieto‐occipital sulcus; (5) Rostral accessory sulcus; (6) Rostral sulcus; (7) Paracalcarine sulcus; (8) Retro‐calcarine sulcus; (A) Rostro of the corpus callosum; (B) Genu of the corpus callosum; (C) Trunk of the corpus callosum; (D) Splenic of the corpus callosum; (E) Cingulate gyrus; (F) Cuneal gyrus; (G) Fornix; (H) Septum pellucidum. (Bar 1 cm).

In the medial part of the brain of *A. belzebul* , the corpus callosum sulcus, the cingulate sulcus, the calcarine sulcus, the parieto‐occipital sulcus, the accessory rostral sulcus and rostral sulcus were identified. These sulci delimit important gyri in the medial region of the brain such as the cingulate gyrus that surrounds the corpus callosum and continues inferiorly with the parahippocampal gyrus, contouring to the diencephalon. The corpus callosum is subdivided into rostro, genu, trunk and splenium of the corpus callosum making the connection between the right and left hemispheres. The structures of the fornix and the pellucidum septum of the diencephalon region of *A. belzebul* were observed (Figure 7).

In *A. belzebul* , the cingulate sulcus was observed in the medial part of the brain, with origin in the anterior part near the rostral sulcus and with ascending path to the superomedial part forming a marginal branch; it presented itself as long, with slight sinuosity and depth. These data corroborate the findings in *S. libidinosus* (Pereira‐de‐Paula et al. 2010), *A. geoffroyi* , *B. arachnoides* , *A. seniculus* (Conolly 1936), *M. mulatta* (Geist 1930), *M. fascicularis* (Kashima et al. 2008; Fukunishi et al. 2006), *P. cynocephalus* and *P. troglodytes* (Conolly 1936; Swindler and Wood 1973). In *G. senegalensis* (Preuss and Goldman‐Raki 1991; Kanagasutheram and Mahran 1960), this sulcus was short with origin in the genu of the corpus callosum and termination before the splenium of the corpus callosum, without originating a marginal branch. In *C. penicillata* (Rylands and Mendes 2008; Abreu et al. 2021), this sulcus is absent. In *Homo*, this sulcus is curved with origin below the rostrum of the corpus callosum; in its posterior path, it is divided into paracentral and marginal branches (Martin 2013; Machado and Haertel 2014; Noureldine 2019) and it is part of the limbic system.

In *A. belzebul* , the rostral sulcus and an accessory rostral sulcus were identified, located in the frontal pole, with a short and shallow path, forming a bifurcation. These data were also verified in *S. libidinosus* (Pereira‐de‐Paula et al. 2010), *A. geoffroyi* , *B. arachnoides* , *A. seniculus* (Conolly 1936), *M. mulatta* (Geist 1930), *M. fascicularis* (Kashima et al. 2008; Fukunishi et al. 2006), *P. cynocephalus* and *P. troglodytes* (Conolly 1936; Swindler and Wood 1973). In *G. senegalensis senegalensis* (Preuss and Goldman‐Raki 1991; Kanagasutheram and Mahran 1960), this sulcus is absent, as well as in *C. penicillata* , and *S. ustus* (Rylands and Mendes 2008; Goldschmidt et al. 2009; Abreu et al. 2021). In *Homo*, this sulcus separates the frontal gyrus into two parts, one superior and one inferior (Testut and Latarjet 1958; Brandão 2004).

In *A. belzebul* , the corpus callosum sulcus has its origin in the rostrum of the corpus callosum, skirting the genu, trunk, splenium and joining with the hippocampus sulcus and the calcarine sulcus in a continuous path. These data corroborate the findings for *S. libidinosus* (Pereira‐de‐Paula et al. 2010), *A. geoffroyi* , *B. arachnoides* , *A. seniculus* (Conolly 1936; León et al. 2009; Mendes et al. 2008), *M. mulatta* (Geist 1930), *Macaca fasciacularis* (Kashima et al. 2008; Fukunishi et al. 2006), *P. cynocephalus* , *P. troglodytes* (Conolly 1936; Swindler and Wood 1973), *G. senegalensis* (Preuss and Goldman‐Raki 1991; Kanagasutheram and Mahran 1960) *C. penicillata* , and *S. ustus* (Rylands and Mendes 2008; Goldschmidt et al. 2009; Abreu et al. 2021). In *Homo*, there is an exception to which the corpus callosum sulcus and the hippocampus sulcus are continuous and are separated from the calcarine sulcus (Noureldine 2019; Ribas 2010; Brandão 2004; Standring 2010).

In Table 1, the main sulci found in *A. belzebul* as well as in other primates from old and new world were demonstrated. The knowledge of the main sulci is important for the delimitation of gyri and lobes, in order to characterise the complexity of the species and provides support for studies of cortical architecture. From the neurophylogenetic point of view, the intelligence of the species has been evaluated by the extension of cortical associative areas, by brain mass and especially by the relation between brain mass/body mass or encephalisation index, the favourite indicator of most anthropologists (Prada 2014). For Martin (1990), one of the characteristics that distinguishes the human species compared to other animals is the size of its telecephalon, constituting an even greater advance within the pattern of large telencephalos of the primates, which, at least in thesis, determines the intelligence.

**TABLE 1:** Main brain sulci of *Alouatta belzebul* compared to other primates.

Cerebral sulci	*Alouatta Belzebul* (Red‐handed howler)	*Galago Senegalensis* (Galago)	*Callithrix penicillata* (Black‐tufted marmoset)	*Callithrix jacchus* (White‐tufted marmoset)	*Saimiri Ustus* (Bare‐eared Squirrel Monkey)	*Sapajus libidinosus* (Capuchin Monkey)	*Alouatta Seniculus* (Jurua Red Howler)	*Ateles geoffroyi* (Ornate Spider Monkey)	*Brachyteles arachnoids* (Woolly spider monkey)	*Macaca Mulatta* (Rhesus monkeys)	*Macaca fascicularis* (Crab‐eating Macaque)	*Papio cynocephalus* (Yellow Baboon)	*Pan troglodytes* (Chimpanzee)	*Homo* (Human)
Central sulcus	X	Absent	Absent	Absent	X	X	X	X	X	X	X	X	X	X
Inferior precentral sulcus (longitudinal)	X	X	X	X	X	X	X	X	X	X	X	X	X	X
Superior precentral sulcus	X	Absent	Absent	Absent	Absent	X	X	X	X	X	X	X	X	X
Postcentral sulcus	X	Absent	Absent	Absent	Absent	X	X	X	X	X	X	X	X	X
Lateral Sulcus	X	X	X	X	X	X	X	X	X	X	X	X	X	X
Straight sulcus (Inferior frontal sulcus)	X	Absent	Absent	Absent	Absent	X	X	X	X	X	X	X	X	X
Superior temporal sulcus	X	Absent	X	X	X	X	X	X	X	X	X	X	X	X
Inferior temporal sulcus	X	Absent	Absent	Absent	Absent	X	X	X	X	X	X	X	X	X
Lunate sulcus	X	Absent	Absent	Absent	X	X	X	X	X	X	X	X	X	X
Inferior occipital sulcus	X	Absent	Absent	Absent	Absent	X	X	X	X	X	X	X	X	X
Cingulate sulcus	X	X	Absent	X	X	X	X	X	X	X	X	X	X	X
Corpus callosum sulcus	X	—	X	X	X	X	—	—	X	X	X	X	X	X
Rostral sulcus	X	Absent	Absent	Absent	Absent	X	X	X	X	X	X	X	X	X
Rostral accessory sulcus	X	—	—	—	—	—	—	—	—	—	—	—	—	—
Subparietal sulcus	Absent	Absent	Absent	Absent	X	X	Absent	Absent	Absent	X	X	X	X	X
Parieto‐occipital sulcus	X	Absent	Absent	Absent	X	X	X	X	X	X	X	X	X	X
Calcarine sulcus	X	X	X	X	X	X	X	X	X	X	X	X	X	X
Calcarine branches	X	Absent	Absent	Absent	X	X	X	X	X	X	X	X	X	X
Occipitotemporal sulcus	X	Absent	Absent	Absent	Absent	X	X	X	Absent	X	X	X	X	X
Hippocampus sulcus	X	X	X	X	X	X	X	X	X	X	X	X	X	X
Collateral sulcus	X	Absent	Absent	Absent	X	X	X	X	X	X	X	X	X	X
Rhinal sulcus	X	X	X	X	X	X	X	X	X	X	X	X	X	X

Numerous other factors can also be used for a comparative analysis on intelligence, such as the relative sizes of the telencephalon and the prefrontal cortex in the telencephalon, the depth of the cerebral cortex sulci, the amount of cortical neurons and the molecular sequences of genes, without, however, being any of them more correct or completely preponderant over the others (Fragaszy et al. 2013). The encephalisation rate could be an objective and indirect way of estimating the intelligence of one species and then comparing it with another. The possible relation between the rate of encephalisation and cognition comes from the verification that a voluminous telencephalon implies a high energy cost and, in primates, it is negatively related to the size of other organs also costly, for example, the intestine (Aiello and Wheeler 1995).

The calculation of the encephalisation index indicated the value of 1.95 for *A. belzebul* . In Table 2, the encephalisation indices between non‐human and human primates can be observed. The *A. belzebul* is phylogenetically closer to *Sapajus* and *Macaca* and farther from species such as *Brachyteles* and *Callithrix*. Then, it is believed that a voluminous telencephalon must be a biological adaptation for primates that have a high rate of encephalisation. If the function of the telencephalon is the processing of information, in the thesis, the greater the volume and the degree of folding of its neocortex, the greater the intelligence of that species. On the other hand, it was observed the presence of several sulci and gyri in *A. belzebul* species, even though it presents lissencephalic characteristics, and the complexity of this species was demonstrated through the analysis of the encephalisation index.

**TABLE 2:** Encephalisation index, mass and dimensions of *Alouatta belzebul* brain compared to other primates.

Species	Mass (g)	Encephalisation Index	Height (mm)	Length (mm)	Width (mm)
*A. belzebul*	50.35	1.95	30.72	60.05	40.91
*Galago*	4.7	—	15	27	21.66
*Sapajus*	71.06	2.12–2.4^b^	36.13	59.95	45.70
*Callithrix*	7	1.7^a^	17.89	29.56	21.43
*Saimiri*	24.12	2.25	28.48	44.38	34.10
*Brachyteles*	122	1.74	47.77	79.51	55.16
*Macaca*	89	2.1^a^	44	72	58
*Homo*	1317	7.4–7.8	—	170–160	140–130

*Note:* Encephalisation index: Routh and Pereira‐de‐Paula.

^a^ Roth and Dicke (2005).

^b^ Pereira‐de‐Paula et al. (2010).

Regarding the cortical anatomical characteristics, an explanation for this great cognitive capacity of primates would be the differences in the morphophysiological factors of the architecture of the cerebral cortex, data that researchers such as Swindler and Wood (1973) cite, that the anatomical differences between the brains of pongids and hominids seem to be qualitative and non‐quantitative, such as cell density.

The measurements of the sulci were made in absolute and relative terms, so that the comparison between *A. belzebul* , *S. libidinosus* and *Homo* could be performed in terms of size and inclination of the main sulci (Tables 3 and 4).

**TABLE 3:** Absolute and relative measurements and inclination of the central sulcus.

Measurements of the extremities of the central sulcus		Means of absolute measurements (cm)			Mean of relative measurements (cm)		
		*Alouatta Belzebul*	*Sapajus libidinosus*	*Homo* *sapiens* ^a^	*A. belzebul*	*S. libidinosus*	*Homo sapiens* ^a^
Superior extremity	To the anterior extremity	2.84	3.06	11.10	0.48	0.48	0.69
	To the posterior extremity	3.21	3.30	4.90	0.54	0.52	0.30
Inferior extremity	To the anterior extremity	2.71	3.20	7.10	0.45	0.50	0.44
	To the posterior extremity	3.18	3.20	8.90	0.53	0.50	0.56
Distance in horizontal projection (inclination)		[(3.18–3.21)–(2.84–2.71)] = 0.16 *Alouatta belzebul* [(3.20–3.30)–(3.06–3.20)] = −0.24 *Sapajus libidinosus* [(8.90–4.90)–(11.10–7.10)] = 8.00 *Homo sapiens*					

^a^ Data obtained from Testut and Latarjet (1958).

**TABLE 4:** Straight and sinuous measurements of the main sulci.

Anatomical sulci	Straight measurements (cm) [1]				Sinusoids measurements (cm) [2]				[1]/[2]		
	RH		LH		RH		LH		*Alouatta Belzebul*	*Sapajus libidinosus*	*Homo sapiens*
	*M*	SD	*M*	SD	*M*	SD	*M*	SD			
Lateral sulcus	3.6	0.30	3.6	0.26	5.3	0.33	5.4	0.55	0.67	0.76	—
Central sulcus	1.10	0.28	1.2	0.23	1.64	0.39	1.8	0.24	0.66	0.97	(9/11.8) = 0.76
Parieto‐occipital sulcus	0.75	0.39	0.8	0.44	1.28	0.66	1.6	0.56	0.54	0.87	—

Abbreviations: LH, left hemisphere; *M*, mean; RH, right hemisphere; SD, standard deviation.

*Source:* Adapted from Pereira‐de‐Paula, 2010.

In relation to the inclination of the central sulcus, the value of 0.16 was observed in *A. belzebul* , in which the superior extremity is posterior to the inferior extremity. Similar data were described in humans, in which the inclination was 8.0 and observed in baboons and chimpanzees (Swindler and Wood 1973), however, in the last two species, they were not measured. For *S. libidinosus* (Pereira‐de‐Paula et al. 2010), the inclination of the central sulcus was −0.24, which demonstrates that the superior extremity is slightly ahead of the inferior.

The relative measurements of the central sulcus extremities in relation to the anterior and the posterior extremities of the brain demonstrate that the central sulcus is located in a relatively posterior position of the *A. belzebul* and *Homo*. In *S. libidinosus* the central sulcus is in the middle portion of the cerebral hemispheres, data indicating that, in proportion, the frontal lobe of *Alouatta* and *Homo* are larger than of *S. libidinosus* . The frontal lobe, more specifically the prefrontal area, was the last part of the cerebral hemispheres to be formed, increases proportionally in the evolutionary scale, reaching maximum development in primates, occupying up to a quarter of the brain mass in humans (Luria 1976). The proportional size of this lobe in *Alouatta* and *Homo* is possibly due to the complex cognitive capacity achieved in these species, in addition to the high complexity of motor functions, also justified by the large extensions occupied by the areas of secondary and tertiary association of the frontal lobe, especially in *Homo* (pre‐motor and prefrontal areas, respectively) (Pereira‐de‐Paula et al. 2010).

The comparative analysis of the brain of primates is fundamentally important in the generation of knowledge about the organisation of the main sulci and gyri; however, it is not able to explain the cognitive and behavioural differences between primates by itself, especially in relation to *A. belzebul* , which has important aspects related to cognition, social behaviour and in this case, to memory, as demonstrated in the degree of sinuosity of the lateral sulcus (Abreu et al. 2021; Aversi‐ferreira et al. 2011; Demes 2011; Tavares and Tomaz 2002; Waga et al. 2006).

The values referring to the degree of sinuosity of the sulci of *A. belzebul* and their comparison with other non‐human and human primates are presented in Table 4.

Regarding the degree of sinuosity, it was found that the lateral sulcus in *A. belzebul* has 0.67, in *S. libidinosus* (Pereira‐de‐Paula et al. 2010) 0.76, in *C. penicillata* 0.89, *B. arachnoides* 0.76 and *S. ustus* 0.77 (Abreu et al. 2021). The data show that the fact that *A. belzebul* presents 0.67 degree of sinuosity of the lateral sulcus characterises an important gyrencephalic region that is directly linked to memory. In other non‐human primate species, no discussion on the degree of sinuosity of the lateral sulcus was presented.

The formation of gyrencephaly, with evident and deep sulci and gyri, responds to a universal physical mechanism that demonstrates how the thickness and the area of the cerebral cortex changes as this organ develops. During this formation, the cortex assumes the most stable configuration according to its surface and bends in response to the forces to which it is subjected during its development, such as the pressure of the cerebrospinal fluid, which pushes it out, and the nerve fibres, which pull it in, thus explaining the formation of the cortexes both gyrencephalic and lissencephalic (Smart and Mcsherry 1986).

The central sulcus has a degree of sinuosity of 0.66 in *A. belzebul* , in Homo 0.76 (Testut and Latarjet 1958) and in *S. libidinosus* was 0.97, as described by Pereira‐de‐Paula et al. (2010), with lissencephalic characteristics. In *Brachyteles*, this sulcus has a degree of sinuosity of 0.84 and in *Saimiri* 1 (Abreu et al. 2021), being a totally straight sulcus in this species. *C. penicillata* *'s* telenocephalon is lissencephalic in the frontal, parietal and occipital regions, which makes it difficult to precisely delimit the cerebral hemispheres in lobes and gyri, and thus the central sulcus has not been described, according to Abreu et al. (2021). In other non‐human primate species, no discussion on the degree of sinuosity of the central sulcus was presented.

The parieto‐occipital sulcus has a degree of sinuosity of 0.54 in *A. belzebul* , with gyrencephalic characteristics, in *S. libidinosus* 0.87 (Pereira‐de‐Paula et al. 2010) and in *Brachyteles* 0.81 (Abreu et al. 2021), separating the parietal and occipital lobes, and in both, with lissencephalic characteristics. In other non‐human primate species, no discussion on the degree of sinuosity of the parieto‐occipital sulcus was presented.

According to Mota and Herculano‐Houzel (2015), it was long believed that the degree of gyrencephaly of the cerebral cortex would be associated with its ability to house more neurons. It was believed that these folds would be consequences of increasing the number of neurons distributed in this region throughout the evolution of mammals and in their results, analysing 74 species of mammals, it was found that the degree of gyrencephaly has nothing to do with the amount of neurons or how they are distributed in this region, but when the cortex is bent at the end of embryonic development, it remains in the most stable physical configuration.

The mathematical model proposed by Bayly et al. (2013) verified that in humans and many mammals the degree of folds of the cortex would be related to its tangential expansion, while the deeper layers would develop in response to the stress caused by this process. If the cortex expands faster, the length of the brain circumvolutions, called gyri, would be shorter and more folded. On the other hand, if this process is inferior, the length of these circumvolutions would be longer and its surface would be smoother.

Understanding the degree of gyrencephaly and/or lissencephalic in non‐human primates is as important as studying the very emergence of consciousness, of high cognition and considering a more evolved species, where recent scientific literature describes that the degrees of gyri and sulci or their smooth surface are related to the thickness and the extension of the cerebral cortex and not only to the amount of neurons in the region (Kaas 2006; Isler and Schaik 2009; Jerison 1977). Thus, a lissencephalic species can, in the thesis, be as intelligent as a gyrencephalic species. The *A. belzebul* species is primarily lissencephalic seen macroscopically, but the quantification of cortical neurons and the mathematical relation between thickness and area of the cortex were not verified in this study. Mota and Herculano‐Houzel (2015) found that the mammal's cortex bends approximately when the total area of the cortex grows a thousand times more than the square of the thickness of the cortex. In this line, the authors were able to explain diseases such as lissencephalic in human primates, a genetic condition that alters the ‘bending’ process of the telencephalon and causes seizures and learning difficulties, in which the cortex becomes thick and with a smaller surface. Due to this mathematical relation resulting from the thickness and the area of the cerebral cortex, as well as the amount of neurons, it is that non‐human primates and inferior lissencephalic mammals do not present these pathological processes such as human primates.

## 4 Conclusion

According to the results obtained, it was found that the brain surface of *A. belzebul* presented with lissencephalic characteristics, data similar to those observed in other primates such *as A. geoffrovi*, *A. seniculus* , *M. fascicularis* , *M. mulatta* , *P. cynocephalus* , *C. penicillata* , *S. ustus* and *B. arachnoides* , differing *from* *S. libidinosus* , *Pan* and *Homo*, which have gyrencephalic characteristics.

Thus, in the brain of *A. belzebul* even though lissencephalic, it was possible to evidence the presence of several sulci and gyri with short and shallow paths, corroborating the sulci present in species of gyrencephalic characteristic, such as *Pan* and *Homo*. These data are important in the anatomical description because the presence of these sulci delimits smaller cortical areas to which they may be involved in cortical architecture and thus infer in the design of the complexity of the species.

Phylogenetically, *A. belzebul* is close to *Sapajus* and *Macaca* and far from species such as *Brachyteles* and *Callithrix*, presenting an encephalisation index of 1.95. Even qualitatively, these data indicate that the species *A. belzebul* presents expressive cognition and intelligence.

## Conflicts of Interest

The authors declare no conflicts of interest.

## Data Availability

The data that support the findings of this study are available on request from the corresponding author. The data are not publicly available due to ethical restrictions.

## References

[ahe70104-bib-0001] Abreu, T. , M. C. H. Tavares , R. Bretas , R. C. Rodrigues , A. Pissinati , and T. A. Aversi‐Ferreira . 2021. “Comparative Anatomy of the Encephalon of New World Primates With Emphasis for the *Sapajus* sp.” PLoS One 16, no. 9: e0256309. 34469439 10.1371/journal.pone.0256309 PMC8409804

[ahe70104-bib-0002] Aiello, L. C. , and P. Wheeler . 1995. “The Expensive‐Tissue Hypothesis.” Current Anthropology 02: 199–221.

[ahe70104-bib-0003] Armstrong, E. , K. Zilles , M. Curtis , and A. Schleicher . 1991. “Cortical Folding, the Lunate Sulcus and the Evolution of the Human Brain.” Journal of Human Evolution 25: 341–348.

[ahe70104-bib-0004] Aversi‐ferreira, T. A. , R. S. Maior , F. O. Carneiro‐e‐Silva , et al. 2011. “Comparative Anatomical Analyses of the Forearm Muscles of *Cebus libidinosus* (Rylands et al. 2000): Manipulatory Behavior and Tool Use.” PLoS One 6, no. 7: 1–8. 10.1371/journal.pone.0022165 PMC3137621 21789230

[ahe70104-bib-0005] Bayly, P. V. , R. J. Okamoto , G. Xu , et al. 2013. “A Cortical Folding Model Incorporating Stress‐Dependent Growth Explains Gyral Wavelengths and Stress Patterns in the Developing Brain.” Physical Biology 10: 1–27. 10.1088/1478-3975/10/1/016005 PMC3616769 23357794

[ahe70104-bib-0006] Brandão, M. L. 2004. As Bases Biológicas do Comportamento: Introdução à Neurociência, 223. Editora Pedagógica e Universitária.

[ahe70104-bib-0007] Connolly, C. J. 1950. External Morphology of the Primate Brain. 1st ed, 386. Charles Thomas Publisher.

[ahe70104-bib-0008] Conolly, C. J. 1936. “The Fissural Pattern of the Primate Brain.” American Journal of Physical Anthropology XXI, no. 3: 301–422.

[ahe70104-bib-0009] Demes, B. 2011. “Three‐Dimensional Kinematics of Capuchin Monkey Bipedalism.” American Journal of Physical Anthropology 145, no. 1: 147–155. 21365612 10.1002/ajpa.21484

[ahe70104-bib-0010] Fragaszy, D. M. , Q. Liu , B. W. Wright , et al. 2013. “Wild Bearded Capuchin Monkeys (*Sapajus libidinosus*) Strategically Place Nutsin a Stable Position During Nut‐Cracking.” PLoS One 8, no. 2: 1–9. 10.1371/journal.pone.0056182 PMC3584076 23460793

[ahe70104-bib-0011] Fragaszy, D. M. , E. Visalberghi , and L. M. Fedigan . 2004. The Complete Capuchin – The Biology of the Genus Cebus. 1st ed, 339. Cambridge University Press.

[ahe70104-bib-0012] Fukunishi, K. , K. Sawada , M. Kashima , H. Sakata‐Haga , K. Fukuzaki , and Y. Fukui . 2006. “Development of Cerebral Sulci and Gyri in Fetuses of Cynomolgus Monkeys ( *Macaca fascicularis* ).” Anatomy and Embryology 211, no. 6: 757–764. 17072644 10.1007/s00429-006-0136-7

[ahe70104-bib-0013] Geist, F. D. 1930. “The Brain of the Rhesus Monkey.” Journal of Comparative Neurology 50, no. 2: 333–375.

[ahe70104-bib-0014] Goldschmidt, B. , A. Mota‐Marinho , C. Araújo‐Lopes , et al. 2009. “Sexual Dimorphism in the Squirrel Monkey, Saimiri Sciureus (Linnaeus, 1758) and Saimiri Ustus (I. Geoffroy, 1844) (Primates, Cebidae).” Brazilian Journal of Biology 69, no. 1: 171–174. 10.1590/s1519-69842009000100022 19347161

[ahe70104-bib-0015] Gregorin, R. 2006. “Taxonomia e Variação Geográfica Das Espécies do Gênero *Alouatta lacépède* (Primates, Atelidae) no Brasil.” Revista Brasileira de Zoologia 23, no. 1: 64–144.

[ahe70104-bib-0016] Isler, K. , and C. P. V. Schaik . 2009. “The Expensive Brain: A Framework for Explaining Evolutionary Changes in Brain Size.” Journal of Human Evolution 57, no. 4: 392–400. 19732937 10.1016/j.jhevol.2009.04.009

[ahe70104-bib-0017] Jerison, H. J. 1977. “The Theory of Encephalisation.” Annals of the New York Academy of Sciences 30: 146–160. 10.1111/j.1749-6632.1977.tb41903.x 280197

[ahe70104-bib-0018] Kaas, J. H. 2006. “Evolution of the Neocortex.” Current Biology 16, no. 21: 91–102. 10.1016/j.cub.2006.09.057 17084684

[ahe70104-bib-0019] Kanagasutheram, R. , and Z. Y. Mahran . 1960. “Onservations on the Nervous System of the Lesser Bush Baby (*Galago senegalensis*).” Journal of Anatomy 94: 512–528. 13751110 PMC1244350

[ahe70104-bib-0020] Kashima, M. , K. Sawada , K. Fukunishi , H. Sakata‐Haga , H. Tokado , and Y. Fukui . 2008. “Development of Cerebral Sulci and Gyri in Fetuses of Cynomolgus Monkeys (*Macaca fascicularis*). II. Gross Observation of the Medial Surface.” Brain Structure and Function 212, no. 6: 513–520. 18236075 10.1007/s00429-008-0171-7

[ahe70104-bib-0021] Kiernan, J. A. 2003. Neuroanatomia Humana de Barr. 7th ed, 518. Manole.

[ahe70104-bib-0022] Klüver, H. , and P. C. Bucy . 1937. “Psychic Blindness and Other Symptoms Following Bilateral Temporal Lobectomy in Rhesus Monkeys.” American Journal of Physiology 119: 352–353.

[ahe70104-bib-0023] León, F. C. P. , D. Platas‐Neri , J. Muñoz‐Delgado , et al. 2009. “Cerebral Anatomy of the Spider Monkey *Ateles geoffroyi* Studied Using Magnetic Resonance Imaging. First Report: A Comparative Study With the Human Brain *Homo sapiens* .” Revista Ciencias de la Salud 7, no. 1: 10–27.

[ahe70104-bib-0024] Luria, A. R. 1976. The Working Brain: An Introduction to Neuropsychology, 400. Penguin Books.

[ahe70104-bib-0025] Machado, A. B. M. 1993. Neuroanatomia Funcional. 2nd ed. Atheneu.

[ahe70104-bib-0026] Machado, A. B. M. , and L. M. Haertel . 2014. Neuroanatomia Funcional. 3rd ed. Atheneu.

[ahe70104-bib-0027] MacLean, P. 1990. The Triune Brain in Evolution. Role in Paleocerebral Functions. Plenum Press. 10.1126/science.250.4978.303-a 17797318

[ahe70104-bib-0028] Markowitsch, H. J. , M. Pritzel , M. Wilson , and I. Divac . 1890. “The Prefrontal Cortex of a Prosimian (*Galago senegalensis*) Defined as the Cortical Projection Area of the Thalamic Mediodorsal Nucleus.” Neuroscience 10, no. 5: 1771–1779. 10.1016/0306-4522(80)90094-9 7432621

[ahe70104-bib-0029] Martin, J. H. 2013. Neuroanatomia: Texto e Atlas, 541. AMGH Editora.

[ahe70104-bib-0030] Martin, R. D. 1990. Primate Origins and Evolution: A Phylogenetic Reconstruction. Chapman and Hall.

[ahe70104-bib-0031] Mendes, S. L. , F. Melo , A. B. Rylands , et al. 2008. “*Brachyteles arachnoides*. The IUCN Red List of Threatened Species.”

[ahe70104-bib-0032] Meneses, M. S. 2016. Neuroanatomia Aplicada. 3rd ed, 351. Grupo Gen‐Guanabara Koogan.

[ahe70104-bib-0033] Milton, K. 1984. “Habitat, Diet and Activity Patterns of Free‐Ranging Woolly Spider Monkeys (*Brachyteles arachnoides*, 1806).” International Journal of Primatology 5, no. 5: 491.

[ahe70104-bib-0034] Mota, B. , and S. Herculano‐Houzel . 2015. “Cortical Folding Scales Universally With Surface Area and Thickness, Not Number of Neurons.” Science 349, no. 6243: 74–77. 26138976 10.1126/science.aaa9101

[ahe70104-bib-0035] Noureldine, M. H. A. 2019. Fundamentos da Neuroanatomia: um Guia Clínico. 1st ed. Elsevier.

[ahe70104-bib-0036] Pereira‐de‐Paula, J. , Y. C. L. Prado , C. Tomaz , et al. 2010. “Anatomical Study of the Main Sulci and Gyri of the *Cebus libidinosus* Brain (Rylands, 2000).” Neurobiologia 73, no. 2: 65–78.

[ahe70104-bib-0037] Platas‐Neri, D. , S. Hidalgo‐Tobón , F. L. Chico‐Ponce , et al. 2019. “Brain Connectivity in *Ateles geoffroyi*: Resting‐State Functional Magnetic Resonance Imaging of Working Memory and Executive Control.” Brain, Behavior and Evolution 93: 19–33. 31039559 10.1159/000499177

[ahe70104-bib-0038] Prada, I. 2014. Neuroanatomia Funcional em Medicina Veterinária com Correlações Clínicas. Terra Molhada.

[ahe70104-bib-0039] Preuss, T. M. , and P. C. Goldman‐Raki . 1991. “Architectonics of the Parietal and Temporal Association Cortex in the Strepsirhine Primate Galago Compared to the Anthropoid Primate Macaca.” Journal of Comparative Neurology 310: 475–506. 1939733 10.1002/cne.903100403

[ahe70104-bib-0040] Redouté, J. , S. Stoléru , M. Pugeat , et al. 2005. “Brain Processing of Visual Sexual Stimuli in Treated and Untreated Hypogonadal Patients.” Psychoneuroendocrinology 30: 461. 15721058 10.1016/j.psyneuen.2004.12.003

[ahe70104-bib-0041] Reis, F. P. , and E. A. Erhart . 1979. “The Brain of the Marmoset (*Callitrhix jacchus*).” Acta Anatomica 103: 350–357. 107717

[ahe70104-bib-0042] Ribas, G. C. 2010. “The Cerebral Sulci and Gyri.” Neurosurgical Focus 28: 1–24. 20121437 10.3171/2009.11.FOCUS09245

[ahe70104-bib-0043] Roth, G. , and U. Dicke . 2005. “Evolution of the Brain and Intelligence.” Trends in Cognitive Sciences 9: 250–257. 15866152 10.1016/j.tics.2005.03.005

[ahe70104-bib-0044] Rylands, A. B. , and S. L. Mendes . 2008. “*Callithrix penicillata*. The IUCN Red List of Threatened Species.”

[ahe70104-bib-0045] Sawada, K. , K. Hikishima , A. Y. Murayama , et al. 2014. “Fetal Sulcation and Gyrification in Common Marmosets (*Callithrix jacchus*) Obtained by Ex Vivo Magnetic Resonance Imaging.” Neuroscience 257: 158–174. 24220690 10.1016/j.neuroscience.2013.10.067

[ahe70104-bib-0046] Smart, M. , and G. M. Mcsherry . 1986. “Gyrus Formation in the Cerebral Cortex in the Ferret. I. Description of the External Changes.” Journal of Anatomy 146: 141–152. 3693054 PMC1166530

[ahe70104-bib-0047] Standring, S. 2010. Gray's Anatomia, 1551. Elsevier.

[ahe70104-bib-0048] Swindler, D. R. , and C. D. Wood . 1973. An Atlas of Primate Gross Anatomy, 368. University of Washington Press.

[ahe70104-bib-0049] Tamraz, J. C. , and Y. G. Comair . 2000. Atlas of Regional Anatomy of the Brain Using MRI With Functional Correlations, 346. Springer.

[ahe70104-bib-0050] Tavares, M. C. H. , and C. A. B. Tomaz . 2002. “Working Memory in Capuchin Monkeys (*Cebus apella*).” Behavioural Brain Research 131, no. 1–2: 131–137. 11844580 10.1016/s0166-4328(01)00368-0

[ahe70104-bib-0051] Testut, L. , and A. Latarjet . 1958. Tratado de Anatomia Humana. 9th ed. Salvat.

[ahe70104-bib-0052] Turner, W. M. 1890. “The Convolutions of the Brain: A Study in Comparative Anatomy.” Journal Anatomy of Physiology 25: 105–153. PMC1328113 17231891

[ahe70104-bib-0053] Van De Graaff, K. M. 2003. Anatomia Humana, 840. Manole.

[ahe70104-bib-0054] Waga, I. C. , A. K. Dacier , P. S. Pinha , and M. C. H. Tavares . 2006. “Spontaneous Tool Use by Wild Capuchin Monkeys ( *Cebus libidinosus* ) in the Cerrado.” Folia Primatologica 77, no. 5: 337–344. 10.1159/000093698 16912501

